# Creating a Rehabilitation Sports Public Service Information Systems Using Service Design Thinking: Physical Activity Management of the Disabled after Discharge in the Republic of Korea

**DOI:** 10.3390/healthcare12050594

**Published:** 2024-03-06

**Authors:** Jiyoung Park, Dongheon Kang, Seon-Deok Eun

**Affiliations:** 1 Department of Safety and Health, Wonkwang University, Iksan 54538, Jeonbuk, Republic of Korea; withji0@wku.ac.kr; 2 Department of Healthcare and Public Health Research, National Rehabilitation Center, Ministry of Health and Welfare, Seoul 01022, Republic of Korea

**Keywords:** people with disabilities, disabled persons, after discharge with disabilities, rehabilitation sports, public health service, information delivery system

## Abstract

The Republic of Korea has established an institutional framework to expedite the provision of rehabilitation sports public services to individuals with disabilities post-hospital discharge (Act on Guarantee of Right to Health and Access to Health Services for People with Disabilities in December 2017). Regrettably, this service remains non-operational to date. This study employs a service design approach to identify and develop essential elements for the effective implementation of public rehabilitation sports services in Korea. Adopting the service design method, including the empathize–define–ideate–prototype phases, co-creation activities were conducted with three teams comprising people with disabilities, caregivers, rehabilitation physicians, specialized sports instructors, facility managers, and government officials, emphasizing equitable distribution. By leveraging the experiences of people with disabilities, these teams collaboratively engaged in creative activities to formulate strategies for delivering prompt and user-friendly rehabilitation sports public services post-hospital discharge. Contributions from each team were meticulously collected and organized, incorporating diverse perspectives into the development of the Korean Rehabilitation Sports Public Service Information System (KRSPSIS). Additionally, we presented a scenario illustrating the practical application of the KRSPSIS. Through this system, we anticipate providing more efficient and convenient rehabilitation sports public services to individuals with disabilities during the critical early stages following hospital discharge.

## 1. Introduction

In the Republic of Korea, as of the end of 2020, 2,633,000 registered individuals, constituting 5.1% of the total population had encountered disability-related challenges following hospital discharge [1]. This escalation in the disabled population is linked to the expanding elderly demographic resulting from super-aging. Furthermore, a surge in risky behaviors and accidents, illustrated by the prevalence of extreme sports, is thought to fuel this rising trend. The inadequacies of immediate support programs and specialized personnel significantly underscore the challenges in health management and adjustment faced by individuals immediately after discharge [2]. Among the registered individuals, 37.4% fall under severe disabilities, leading to escalating social and economic challenges, including heightened medical expenses. The average annual medical expenses per person, particularly hospitalization and outpatient treatment fees, are substantially higher for individuals with disabilities, peaking in cases of brain disabilities (such as stroke). Additionally, projections indicate the healthcare spending share in GDP (gross domestic product) reaching 10.2% by 2023 in the Republic of Korea, with an average annual growth rate of 5% (2019–2023), significantly surpassing the previous rate of 2.7% (2014–2018) [3,4].

The World Health Organization (WHO) [5] underscores the vulnerability of individuals with disabilities to inadequate health maintenance, leading to a higher risk of additional health problems and complications, ultimately reducing life expectancy [6,7]. Health check-up data from the National Health Insurance Service of the Republic of Korea indicate that over 65.1% of individuals with disabilities do not engage in physical activities [8]. Regular physical activity is known to enhance cardiopulmonary function, muscle strength, and reduce the incidence of cardiovascular and chronic diseases. For individuals with disabilities, participation in physical activity is vital for preserving independent function, aiding in recovery, and preventing secondary health issues [9,10,11].

In the Republic of Korea, there is a disability sports program organized by the Korea Paralympic Committee under the Ministry of Culture, Sports, and Tourism. Engaging in sports activities focused on specific disciplines for individuals with disabilities is challenging for them immediately after discharge from the hospital. Regular physical activity for individuals with disabilities is beneficial for maintaining independent functionality and aiding recovery. However, it is challenging for individuals to initiate such activities on their own, especially in the early stages of discharge. People with disabilities who have not participated in physical activities in the early stages of discharge tend to be less likely to engage in sports activities later on. Diminished physical activity due to disabilities is a major contributor to increased healthcare costs.

In an effort to mitigate this vicious cycle, the Ministry of Health and Welfare implemented the ‘Act on Guarantee of Right to Health and Access to Health Services for PWD (hereinafter ‘PWD Health Rights Act’)’ in December 2017. The Ministry of Health and Welfare, in collaboration with the National Rehabilitation Center, has established a consultative body for rehabilitation sports, involving experts from disability organizations, the Ministry of Culture, Sports, and Tourism, relevant agencies, and academia. Through this collaboration, they are discussing plans for delivering useful and effective public services. For the successful provision of rehabilitation sports public services, effective collaboration is crucial among various stakeholders, including the Ministry of Health and Welfare, the Ministry of Culture, Sports, and Tourism; sports facility managers; rehabilitation sports instructors; disability organizations; and social workers. Seamless communication among these stakeholders is essential.

The aim of this study is to propose methods for the smooth delivery of rehabilitation sports public services to individuals with disabilities in the early stages of discharge, utilizing service design approaches.

## 2. Methods

The delivery system for rehabilitation sports public services developed in this study is intended to be utilized in public services provided by the Ministry of Health and Welfare, targeting people who have acquired disabilities in their daily lives. Public services provided by the government are primarily evaluated based on citizen satisfaction. Cornwall and Coelho [12] have emphasized the importance of involving citizens in the critical decision-making processes of public institutions to enhance public service satisfaction. They underscored the need for the government to promptly respond to the demands and voices of the public in order to elevate satisfaction levels.

The concept of design thinking is gaining prominence as a method to strengthen social and public policies and address issues related to innovation. Design thinking offers broad and applicable alternatives, optimizing universality through the iterative verification of solutions derived from the needs of minority populations [13]. Additionally, design thinking possesses the strength of fostering a shared understanding of proposals generated by teams across various fields [14], making it a valuable approach for addressing diverse challenges and promoting inclusive problem solving. It has been adopted and applied by numerous international public institutions, including the UK Design Council, Finland’s Finnish Innovation Fund (SITRA), and The Australian Centre for Social Innovation (TACSI). Design Thinking emphasizes the creative problem-solving abilities of designers as they strive to balance intuitive and analytical thinking [15,16]. Successful applications of design thinking include providing innovative solutions for policy environment management [17] and addressing the complexity of user demands [18], demonstrating the effectiveness of design thinking methodologies through such cases.

**Figure 1:**
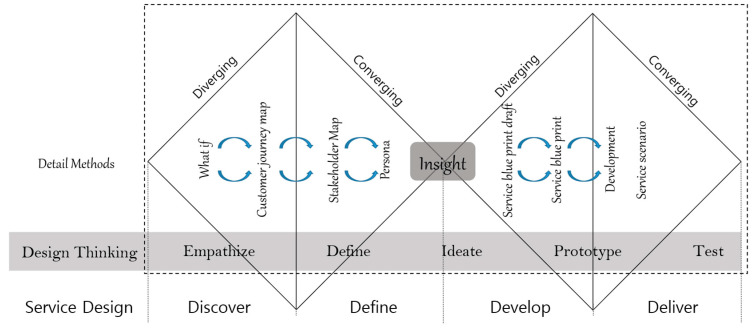
Step-by-step design thinking methodology for developing the KRSPSIS.

To enhance healthcare services effectively, it is imperative to conduct experience-based collaborative research [19]. Service design methods are actively employed in this type of research, focusing on identifying novel service demands, expectations, and experiences from diverse stakeholders [20]. The integration of design thinking methodology in service design has found application in public service industries such as education, transportation, and healthcare. Its success in government projects has garnered recognition from both academic scholars and design practitioners [21]. Unlike products with tangible affordances, services can be abstract or invisible. While quantitative methods are useful for understanding a particular field, they may not be effective for tasks that involve consideration of genuine personal relationships. Service design methods, such as qualitative research approaches, are effective for service development that involves a blend of management, access, and response based on insights derived from individual experiences. By focusing on how services operate from an individual perspective, service design methods enable the discovery of greater overall value, facilitating the achievement of outcomes that consider and prioritize genuine personal relationships [22]. Generally, service design aims to redefine customer experiences through a four-stage process, encompassing discovery, definition, development, and delivery. This process is characterized by iterative cycles of divergent and convergent thinking (Figure 1). The 4D process is comprised of the stages discover, define, develop, and deliver, encompassing the following: 1st stage—empathize (understanding user needs); 2nd stage—define (clarifying user needs and problems); 3rd stage—ideate (challenging assumptions and generating ideas); 4th stage—prototype (initiating solution creation); and 5th stage—test (evaluating solutions) [23,24,25]. In this study, the methodology illustrated in Figure 1 was employed to implement service design using design thinking.

This study employed various techniques in the service design process:

Brainstorming: Used in workshops to foster creative thinking and consensus building among stakeholders by facilitating the free expression of ideas.

‘What If’ Analysis: Conducted prior to alternative selection, simulating potential result changes based on variations in decision variables, constraints, and the decision environment. A pre-distributed questionnaire compiled individual opinions for subsequent group discussions.

Affinity Map: Grouped and classified ideas on sticky notes to identify patterns and core concepts. Sticky notes addressing issues from ‘What If’ discussions were further grouped and discussed.

Customer Journey Map: Defined a customer’s service experience, visualizing satisfaction changes over stages. Team members documented changes in places, encounters, and emotions over time, fostering understanding and empathy for individuals with disabilities.

Stakeholder Map: Systematically organized stakeholders influencing or influenced by the service, visually representing communication relationships. Identified workforce composition needed for implementing rehabilitation sports services.

Persona: Humanized user groups, guiding focused discussions. Teams selected a representative user type, detailing age, family, hobbies, and goals for rehabilitation sports.

Service Blueprint: An illustrative methodology depicting the entire service process, distinguishing visible and invisible aspects. Visualized the service process, identifying potential issues and areas for improvement.

Prototype: A tangible representation used to reconstruct and enhance a service.

Service Scenario: Provided situations for service concepts and prototypes to aid understanding, enabling anticipation of how people will use new services.

### Participants

**Figure 2:**
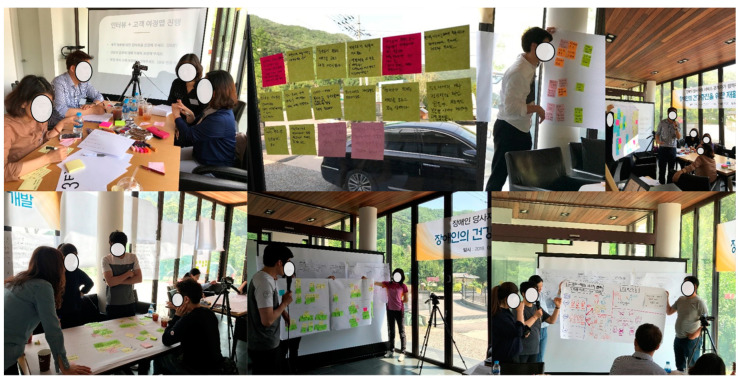
Co-creation to develop the KRSPSIS.

This study was conducted by evenly distributing participants, including people with disabilities, caregivers, doctors, sports facility managers, sports instructors, and government officials, into three teams. A total of 19 participants took part, and the composition of each team is shown in Table 1. People with disabilities and caregivers shared challenges related to physical activity following the onset of disabilities. Each team employed a step-by-step design thinking methodology (refer to Figure 1 and Figure 2) to comprehensively address these issues. The outcomes from the three teams were integrated into the proposed KRSPSIS.

## 3. Results

The outcomes of the service design process employing the design thinking method (Figure 1) are detailed below. After presenting the outcomes individually from each of the three teams, the content was revised through discussion and consolidated into a single result.

### 3.1. Empathize

**Figure 3:**
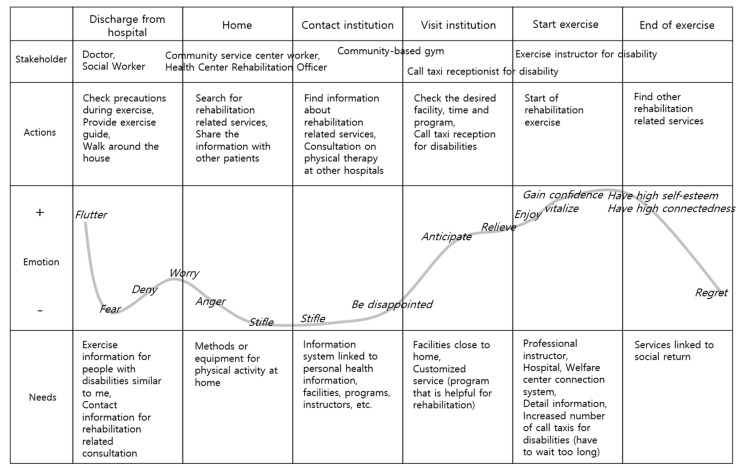
Customer journey map for people with disabilities regarding their exercise processes after hospital discharge.

In the ‘What If’ analysis, challenges in rehabilitation sports services provision were identified, involving difficulties in accessing information, facility accessibility, program availability, instructor shortages, and financial burdens. The customer journey map (Figure 3) revealed a range of emotions post-hospital discharge, including joy and anxiety. Challenges included difficulties in finding suitable exercise services, leading to dissatisfaction with extended wait times and limited benefits. Disabled participants expressed the positive impact of starting exercise on vitality, self-esteem, and confidence, but faced disappointment when unable to sustain exercise due to program discontinuation or facility unavailability.

### 3.2. Define

**Figure 4:**
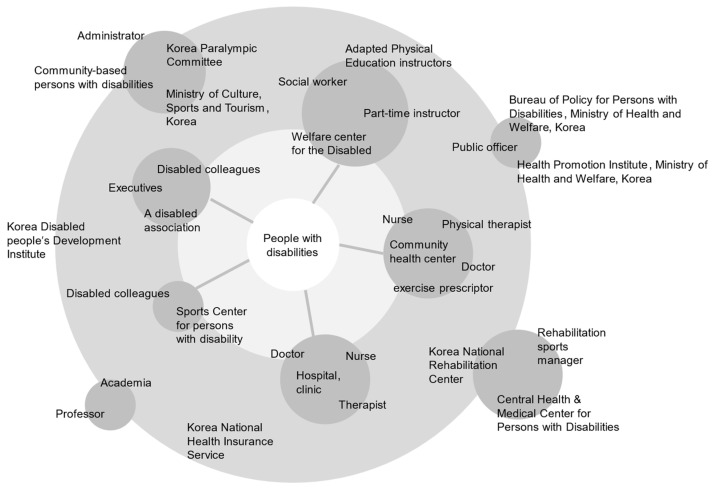
Result of rehabilitation sports services stakeholder’s map.

This study conducted stakeholder analysis (Figure 4) to optimize the efficiency of rehabilitation sports services, focusing on individuals with disabilities. Participants expressed a preference for accessing services through various channels such as PCs, mobile devices, hotlines, and in-person visits. They highlighted the need for consultants to address rehabilitation exercise and sports-related programs, facilities, instructors, and costs. Collaboration between the Ministry of Health and Welfare and the Ministry of Culture, Sports, and Tourism was deemed essential for facility and instructor coordination and post-service engagement. The early assessment of participants’ physical abilities requires medical opinions, but it was suggested that medical opinions should be limited to the confirmation of exercise capabilities during the initial stages of rehabilitation. The creation of personas provided a detailed understanding of the target audience, primarily focusing on individuals with spinal cord and brain lesions, representing the largest segment of the disabled population.

### 3.3. Ideate

**Figure 5:**
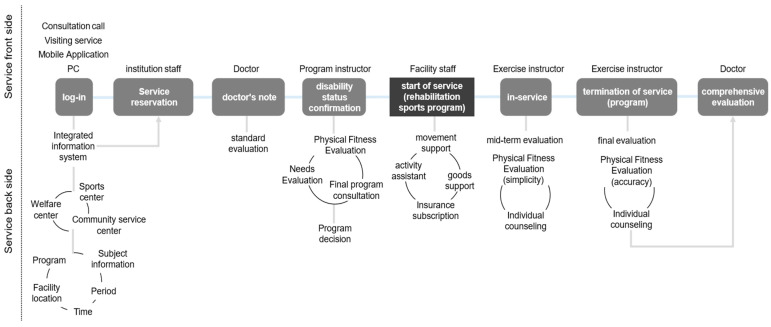
Service blueprint for the KRSPSIS.

In the ideation stage, participants emphasized the necessity of an information system that integrates rehabilitation sports programs, instructors, facilities, and the personal data of individuals with disabilities. This led to the creation of a service blueprint (Figure 5) for virtual personas, enabling the analysis of associated components and service delivery prerequisites prior to the initial contact in rehabilitation sports services.

### 3.4. Prototype

**Figure 6:**
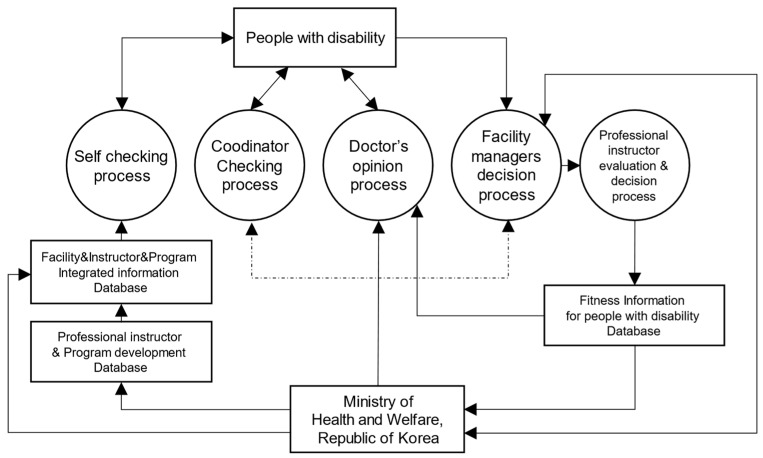
Rehabilitation sports public service delivery system concept.

**Figure 7:**
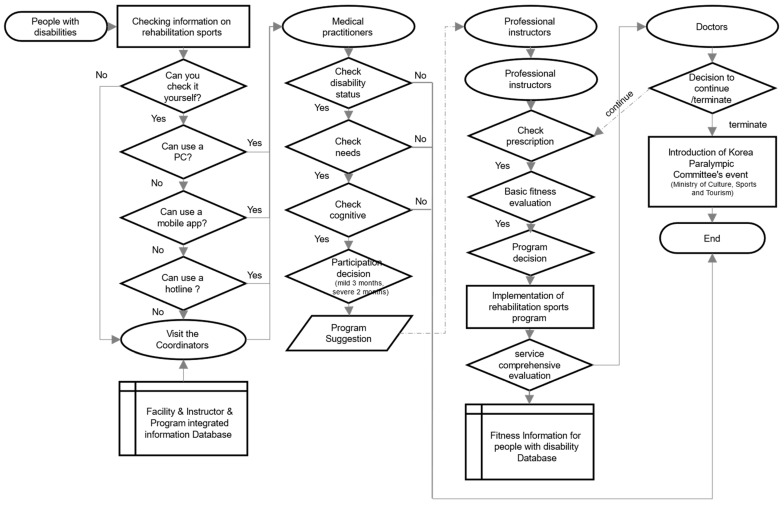
KRSPSIS Prototype.

The service blueprint illustrated in Figure 5 serves as the foundation for our recommended concept of a rehabilitation sports public service delivery system, as depicted in Figure 6. This conceptual model aims to enhance the efficiency and effectiveness of rehabilitation sports services. To complement these conceptual frameworks, Figure 7 introduces a proposed information system concept, synthesizing the elements from Figure 5 and Figure 6. This information system, along with the corresponding roles refined through the incorporation of additional feedback, has resulted in a converged consensus among participants (refer to Table 2 for details). The iterative refinement process, guided by additional feedback, has led to a converged consensus on the feasibility of the proposed system.

The sequential depiction of Figure 5, Figure 6 and Figure 7 provides a comprehensive visualization of the proposed rehabilitation sports public service delivery system and its associated information structure. These figures collectively serve as a foundational framework for understanding and implementing an integrated and efficient rehabilitation sports public service. 

### 3.5. Service Scenario for Use of the KRSPSIS

Step-by-Step Process for Applying for Rehabilitation Sports Services:

Step 1. Initial Eligibility Check: People with disabilities seeking rehabilitation sports services confirm their eligibility as potential users, including those expecting disabilities post-hospitalization or within 3 years of registration.

Step 2. Facility and Program Exploration: Explore desired facilities, service schedules, and programs considering mobility factors through channels like hotlines, mobile apps, or in-person visits. Consult with coordinators for inquiries and assistance.

Step 3. Medical Prescription for Service Application: Visit institutions capable of issuing medical prescriptions for rehabilitation sports services. After counseling and evaluation, receive a medical prescription determining the service duration based on the severity of the disability (2 months for severe, 3 months for mild).

Step 4. Service Application and Additional Recommendations: Apply for services directly at recommended facilities using various means (PC, hotline, mobile app, in-person visits) or revisit the Central Health and Medical Center for additional recommendations.

Step 5. Facility Visit and Program Decision: Visit the chosen facility, consult with instructors, and make informed decisions regarding the selected program.

Step 6. Evaluation and Counseling for Service Continuation: Assess basic fitness and consult with instructors at the program’s completion. Receive guidance on potential integration with leisure sports programs.

Step 7. Final Confirmation with Medical Professional: Meet with a medical professional to make the final decision on the continuation of rehabilitation sports services.

## 4. Discussion

This study employed service design incorporating the 4D process and various design thinking methods to propose the Korean Rehabilitation Sports Public Service Information System (KRSPSIS). The objective was to enhance the efficiency and effectiveness of rehabilitation sports services, aligning with the ‘PWD Health Rights Act’ enforced by the Republic of Korea government in December 2017. Utilizing service design methods, this research aims to identify distinctive service demands and experiences from a diverse range of stakeholders. The integration of design thinking in service design, widely recognized in public service sectors, has proven effective in addressing intangible services. In contrast to quantitative methods, the qualitative approach of service design excels in developing services that consider personal relationships, thereby fostering greater overall value by prioritizing individual perspectives.

In the Republic of Korea, individuals in the early stages of disability often face readmission to hospitals or engage in limited post-discharge physical activities due to the lack of a comprehensive rehabilitation sports service system. This limitation results in restricted access to suitable physical activities aligned with their disabilities and physical capabilities.

We investigated disability sport policies and services in Germany, Japan, the US, the UK, Australia, and France [26,27,28,29,30,31,32,33,34,35,36,37,38]. In Germany, rehabilitation sports for individuals with disabilities are explicitly defined under Social Security Law, Article IX, Paragraph 44–45, and have been implemented since 2011 through agreements on rehabilitation sports and functional training. The emphasis in rehabilitation sports is primarily on group exercises, fostering mutual interaction and support among individuals with disabilities, thereby facilitating rehabilitation and social reintegration.

Japan initially acknowledged the promotion of disabled sports in the Sports Basic Law of 2003, highlighting the need for accommodations to facilitate the voluntary and active engagement of people with disabilities in sports activities in the 2011 Sports Basic Law. Various plans and programs have been established to boost disabled sports, with a focus on encouraging community outreach.

In the United States, grounded in the Americans with Disabilities Act and Rehabilitation Act Section 504, active measures are taken to prohibit discrimination against people with disabilities and support disabled sports activities. The country promotes regular physical activity for individuals with disabilities through the 2008 Physical Activity Guidelines, employing diverse programs to enhance health and activity opportunities for this demographic.

The United Kingdom, guided by anti-discrimination laws, prohibits discrimination against people with disabilities. The UK Disability Sports Association’s “Get Out Get Active (GOGA)” program offers an integrated initiative catering to both disabled and non-disabled individuals. Furthermore, the “Get Yourself Active” initiative empowers individuals with disabilities to autonomously organize and participate in various physical activities.

Australia, through the implementation of the National Disability Insurance Scheme (NDIS) in 2013, laid the groundwork for disability insurance specifically targeting severe disabilities. Disability Sports Australia oversees and supports disabled sports, fostering an environment for individuals with disabilities to partake in local sports activities and advance as elite athletes.

In France, collaborative efforts with sports federations and the Olympic Committee are leveraged to promote disabled sports. The country actively supports disabled sports schools and orchestrates diverse events and programs to amplify the visibility and participation of disabled individuals in sports.

These nations deploy a range of policies, laws, programs, and associations to champion disabled sports, aiming to provide diverse opportunities for individuals with disabilities to participate in various sports and physical activities, ultimately enhancing their overall quality of life.

The findings underscore the significant role of rehabilitation sports in promoting the holistic development of individuals with disabilities, providing emotional fulfillment, and facilitating social integration [39]. These activities empower people with disabilities to improve their quality of life by enhancing physical capabilities and promoting a more normalized daily existence [40]. Noh and Lee [39] highlight the instrumental role of post-therapy or hospital treatment rehabilitation sports in restoring physical and emotional functions, boosting life motivation, and aiding individuals in overcoming life’s challenges.

In the Republic of Korea, the establishment of professional rehabilitation sports services has gained recognition, particularly following the implementation of the PWD Health Rights Act in 2017. However, a gap exists in delivering physical activity services during the early discharge stages from hospitals, necessitating the creation of an information system like the Korean Rehabilitation Sports Public Service Information System (KRSPSIS). Essential components of this process include budget allocation and the recruitment of qualified personnel.

This study delineates the development, content, and utilization scenarios of the KRSPSIS, leveraging the service design method employed in public service development in countries like the UK and the US. The inclusion of perspectives from people with disabilities, doctors, facility managers, academic experts, and government officials is vital for the effectiveness of rehabilitation sports public services.

Concerns raised by individuals with disabilities regarding the lack of exercise recommendations from doctors upon discharge underscore a significant demand for comprehensive prescriptions in the Republic of Korea. To address these gaps, this study utilizes the design thinking method to conceptualize an information system and coordinator arrangement.

Successful KRSPSIS implementation requires various measures, including increasing awareness among people with disabilities, developing tailored programs, training specialized instructors, expanding facilities, ensuring accessibility, and fostering collaboration between relevant ministries [41]. This study acknowledges limitations in the classification of disabilities in Korea, primarily focusing on spinal cord injuries or brain lesions. Future research should encompass various disability types, developing tailored programs and assistive devices for each category, and incorporating standardized assessment items to ensure the KRSPSIS’s data reliability.

## 5. Conclusions

In summary, rehabilitation sports services act as a crucial link connecting medical rehabilitation and leisure sports, facilitating the seamless reintegration of individuals with disabilities into daily life post-hospital discharge. The introduction of the Rehabilitation Sports Public Service holds promise in enhancing the rehabilitation journey by improving physical function, promoting voluntary sports participation, and bolstering psychological well-being. With the envisioned KRSPSIS playing a pivotal role in delivering continuous physical activity services during the early post-discharge stages, our objective is to instill a sense of personal responsibility for healthcare through early engagement in physical activity. We anticipate positive outcomes from these efforts, envisioning a reduction in socioeconomic costs associated with complications from chronic diseases and disabilities.

Moreover, emphasizing the vital collaboration between the Ministry of Health and Welfare and the Ministry of Culture, Sports, and Tourism is essential for the effective utilization of the KRSPSIS. The systematic implementation of the KRSPSIS requires close cooperation between these ministries, contributing to the establishment of an efficient system. This system is poised to empower individuals with disabilities in the Republic of Korea to transition from rehabilitation sports to leisure sports programs, ensuring sustained active participation in physical activities.

## Figures and Tables

**Table 1:** Participants.

A (*n* = 7)	B (*n* = 6)	C (*n* = 6)
Individuals with spinal cord injury (SCI) Caregiver Instructor Sports facility manager Professor of adaptive physical education Representative from the Korean Paralympic Committee Official from the Ministry of Health and Welfare	Individuals with spinal cord injury (SCI) Instructor Two officials from the Ministry of Health and Welfare Sports facility managers Professor of adaptive physical education	Individuals who have experienced a stroke Caregiver Instructor Two officials from the ministry of Health and Welfare Doctor

**Table 2:** Roles for each component.

Name	Roles
Ministry of Health and Welfare (MHW)	Formulates policies for rehabilitation sports services. Fosters cooperation, secures budgets, and manages an integrated information system.
National Health Insurance Service (NHIS)	Allocates budgets, assesses fees, and manages financial resources for people with disabilities. Addresses challenges in budget allocation.
People with disabilities	Focus on those with spinal cord disorders and brain lesions. Access ‘rehabilitation sports services’ information through the KRSPSIS via various means. Apply for programs based on comprehensive information. A follow-up study on various types of disability is required.
Coordinators	Furnish explanations and make recommendations using the KRSPSIS. Consider disability type, severity, residence proximity, and specific requirements. Verify and disseminate information regarding service availability, expenses, and prerequisites.
Doctors	Specialize in ‘rehabilitation sports services’. Recommend suitable programs, assess needs, and conduct cognitive assessments. Provide guidance on service initiation, termination, and continuation. Collect data during counseling sessions.
Medical prescription	Standardized form with essential disability information. Requires subsequent development post-study.
Professional instructors	Customize services for disabilities, evaluate recommendations, and perform assessments. Suggest program duration and introduce leisure sports. Certification requires a new qualification system or continuous education.
Facility-, instructor-, and program- integrated information database	Disseminates information on rehabilitation sports programs, instructors, and facilities. Requires further database and network construction.
Fitness information for people with disability database	Contains participants’ disability status and fitness information. Accesses and inputs rights restricted to doctors and instructors. Further deliberation on information access rights is warranted.
Central Health and Medical Center	Conducts health assessments and delivers healthcare services. Manages health needs and collaborates with local centers for comprehensive management.

## Data Availability

The data presented in this study are available on request from the corresponding author.
